# MRI‐visible perivascular space volume: An in vivo marker of post‐mortem vascular pathology

**DOI:** 10.1002/alz.095526

**Published:** 2025-01-09

**Authors:** Lisa C Silbert, David Lahna, Daniel Schwartz, Natalie E Roese, Justin Hurworth, Rachel C Wall, Victoria Krajbich, Randall Woltjer

**Affiliations:** ^1^ Portland Veterans Affairs Medical Center, Portland, OR USA; ^2^ NIA‐Layton Oregon Alzheimer’s Disease Research Center, Oregon Health & Science University, Portland, OR USA; ^3^ Advanced Imaging Research Center, Oregon Health & Science University, Portland, OR USA

## Abstract

**Background:**

MR‐visible perivascular space (PVS) burden is associated with clinical and MRI features of cerebrovascular disease. Its utility as an in vivo biomarker of post‐mortem pathology is uncertain.

**Method:**

Eighteen older adults (age at death 98.0, sd 5.5; 83% female) with autopsy consent were followed longitudinally in the Oregon Alzheimer’s Disease Research Center with MRI and cognitive assessments until death. In vivo MR‐visible white matter (WM) PVS volumes were derived using an automated segmentation algorithm based on T1 intensity thresholding and morphological constraints. Basal ganglia (BG) PVS were hand‐traced on a single axial T1 slice with highest number of PVS. WM and BG PVS volumes were summed and normalized to brain volume to create a PVS score. General linear models (GLM) examined relationships between whole brain PVS score and: 1) cognition, and 2) post‐mortem vascular pathologies. Models were adjusted for age, sex, and time between MRI and death when applicable.

**Result:**

In vivo MRI PVS score was associated with greater post‐mortem deep microvascular lesions (p = 0.002), arteriolosclerosis (p = 0.004), and atherosclerosis (p = 0.002). In separate models, PVS score was associated with worse performance on Trails B (p = 0.006) and MMSE (p = 0.02), but not memory (p = 0.6).

**Conclusion:**

Greater whole brain in vivo PVS burden is associated with worse cognitive performance in domains commonly associated with cerebrovascular disease and multiple post‐mortem cerebrovascular pathologies. Findings support the use of in vivo PVS as a candidate “V” marker in A/T/N classifications of older individuals

